# Supplementary material for: Estimation of the case fatality rate based on stratification for the COVID-19 outbreak
Source: PLoS One. 2021 Feb 22;16(2):e0246921. doi: 10.1371/journal.pone.0246921 (PMC7899354; doi:10.1371/journal.pone.0246921)
Supplement: S1 Appendix — (PDF) [file pone.0246921.s002.pdf]

S1 Appendix

Additional observation for the proposed estimator

For the proposed estimator of the case fatality rate up to time t, we have the following observation:

Theorem 1. *For each stratum l, $l = 1, \ldots, L$, and given $N_l(t)$, assume $(C_l(t), D_l(t), U_l(t)) \sim \text{Multinomial}(N_l(t), (p_c(l,t), p_d(l,t), p_u(l,t)))$, and $p_u(l,t) \to 0$ as $t \to \infty$. Then,*

(i) $E(\widehat{\text{FR}}_l(t)) = p_d(l,t) + p_d(l,t)p_u(l,t)\frac{N_l(t)-1}{N_l(t)}$, *and* $\widehat{\text{FR}}_l(t)$ $(l = 1, \ldots, L)$ *is asymptotically unbiased as* $t \to \infty$.

(ii) $Var(\widehat{\text{FR}}_l(t)) = \frac{1}{N_l(t)}\eta_l(t) + \frac{1}{N_l^2(t)}\zeta_l(t)$, *where (dropping (l,t) temporarily)*

$$\eta_l(t) = p_d(1-p_d) + \frac{N_l(t)-1}{N_l(t)} p_d p_u \{2 - 3p_d + p_u - 4p_d p_u\},$$

and

$$\zeta_l(t) = \frac{N_l(t)-1}{N_l(t)} p_d p_u \{1 - 2p_d + 6p_d p_u - 2p_u\}.$$

Theorem 2. *Let the assumptions in Theorem 1 hold and assume that* $(C_l(t), D_l(t), U_l(t))$ *and* $(C_\ell(t), D_\ell(t), U_\ell(t))$ *are independent for* $l \neq \ell$. *Then,* $\widehat{\mathrm{FR}}(t)$ *is asymptotically unbiased as* $t \to \infty$, *and*

$$Var(\widehat{\mathrm{FR}}(t)) = \sum_{l=1}^{L} \left(\frac{N_l(t)}{N(t)} \right)^2 \left\{ \frac{1}{N_l(t)} \eta_l(t) + \frac{1}{N_l^2(t)} \zeta_l(t) \right\}.$$

Note that as $t \to \infty$, $p_u(l,t) \to 0$ and $Var(\widehat{\mathrm{FR}}_l(t)) \to \frac{p_d(1-p_d)}{N_l(\infty)}$. If $N_l(t)$ increases as well, then the second term of $Var(\widehat{\mathrm{FR}}_l(t))$ becomes negligible as well.

Proof. For $l = 1, \ldots, L$, we assume

$$(C_l(t), D_l(t), U_l(t)) \sim \text{Multinomial}(N_l(t), (p_c(l,t), p_d(l,t), p_u(l,t))).$$

In this proof, let us drop (t) and (l,t) for ease of presentation. Then, we have the following:

$$\begin{aligned} E(D_l) &= N_l p_d(l), \\ E(D_l U_l) &= N_l(N_l - 1) p_d p_u, \\ E(D_l^2) &= N_l(N_l - 1) p_d^2 + N_l p_d, \\ E(D_l^2 U_l) &= N_l(N_l - 1)(N_l - 2) p_d^2 p_u + N_l(N_l - 1) p_d p_u, \\ E(D_l^2 U_l^2) &= N_l(N_l - 1)(N_l - 2)(N_l - 3) p_d^2 p_u^2 \\ &\quad + N_l(N_l - 1)(N_l - 2)(p_d^2 p_u + p_d p_u^2) + N_l(N_l - 1) p_d p_u. \end{aligned}$$

From the above expected values, we have

(i) $E(\widehat{\mathrm{FR}}_l(t)) = \frac{1}{n} E(D_l + \frac{D_l U_l}{N_l}) = \frac{1}{N_l}(N_l p_d + (N_l - 1) p_d p_u) = p_d + \frac{N_l - 1}{N_l} p_d p_u$. Since we assume that $p_u(l,t) \to 0$ as $t \to \infty$, $\widehat{\mathrm{FR}}_l(t) \to p_d(l, \infty)$ as $t \to \infty$. And we also have

(ii)

$$\begin{aligned} Var(\widehat{\mathrm{FR}}_l(t)) &= E(\widehat{\mathrm{FR}}_l^2(t)) - \{E(\widehat{\mathrm{FR}}_l(t))\}^2, \\ N_l^2 Var(\widehat{\mathrm{FR}}_l(t)) &= E\left(D_l^2 + \frac{2}{N_l} D_l^2 U_l + \frac{D_l^2 U_l^2}{N_l^2} \right) \\ &\quad - \{N_l^2 p_d^2 + 2N_l(N_l - 1) p_d^2 p_u + (N_l - 1)^2 p_d^2 p_u^2\}. \end{aligned}$$

The right hand side is summarized as $\xi_1 + \xi_2$, where

$$\xi_1 = N_l p_d(1 - p_d) + (N_l - 1) p_d p_u (2 - 3p_d + p_u - 4 p_d p_u)$$

and

$$\xi_2 = \frac{N_l - 1}{N_l} p_d p_u (1 - 2p_d + 6 p_d p_u - 2p_u).$$

The proof for Theorem 2 is omitted.
