# Supplementary material for: Estimation of the case fatality rate based on stratification for the COVID-19 outbreak
Source: PLoS One. 2021 Feb 22;16(2):e0246921. doi: 10.1371/journal.pone.0246921 (PMC7899354; doi:10.1371/journal.pone.0246921)
Supplement: Attachment — Submitted filename: Response_to_Reviewers.pdf [file pone.0246921.s003.pdf]

Revision Report (PONE-D-20-13762)

Byungwon Kim, Seonghong Kim, Woncheol Jang, Sungkyu Jung, and Johan Lim

August 3, 2020

We thank the Academic Editor and the three anonymous referees for their thoughtful comments on our manuscript, titled "Estimation of the case fatality rate based on stratification for the COVID-19 outbreak". In the revision, we have followed all of the suggestions except for few cases as explained below, and also have made several improvements. In the following, please find our point-by-point responses to the journal's requirements and referees' comments.

Responses to journal's requirements and questions

Journal Requirements: When submitting your revision, we need you to address these additional requirements.

(1) Please ensure that your manuscript meets PLOS ONE's style requirements, including those for file naming. The PLOS ONE style templates can be found at `https://journals.plos.org/plosone/s/file?id=wjVg/PLOSOne_formatting_sample_main_body.pdf` and `https://journals.plos.org/plosone/s/file?id=ba62/PLOSOne_formatting_sample_title_authors_affiliations.pdf`

Answer: We have carefully checked the PLOS ONE's style requirements and revised the manuscript accordingly.

Reviewer's Responses to Questions:

(2) Is the manuscript technically sound, and do the data support the conclusions?

The manuscript must describe a technically sound piece of scientific research with data that supports the conclusions. Experiments must have been conducted rigorously, with appropriate controls, replication,

and sample sizes. The conclusions must be drawn appropriately based on the data presented.

Reviewer #1: Partly

Reviewer #2: Yes

Reviewer #3: Yes

Answer: In this manuscript, we propose a new estimator of the case fatality rate for a communicable disease and provide COVID-19 cases in China, South Korea, and the United States for real world examples. The main topic of this manuscript is not related with a experimental study, instead, the conclusions provided in the manuscript are carefully derived from the observations in COVID-19 cases.

(3) Has the statistical analysis been performed appropriately and rigorously?

Reviewer #1: N/A

Reviewer #2: Yes

Reviewer #3: Yes

Answer: In the numerical examples (China, South Korea, and the United States), the case fatality rate (CFR) is estimated using the proposed estimator on a daily basis and corresponding confidence interval is also provided, which is obtained based on the variance of the proposed estimator and large sample approximation.

(4) Have the authors made all data underlying the findings in their manuscript fully available?

The PLOS Data policy requires authors to make all data underlying the findings described in their manuscript fully available without restriction, with rare exception (please refer to the Data Availability Statement in the manuscript PDF file). The data should be provided as part of the manuscript or its supporting information, or deposited to a public repository. For example, in addition to summary statistics, the data points behind means, medians and variance measures should be available. If there are restrictions on publicly sharing datae.g. participant privacy or use of data from a third partythose must be specified.

Reviewer #1: Yes

Reviewer #2: Yes

Reviewer #3: No

Answer: We post all data used in the manuscript on our website. The address is provided in the Supporting Information section.

(5) Is the manuscript presented in an intelligible fashion and written in standard English?

PLOS ONE does not copyedit accepted manuscripts, so the language in submitted articles must be clear, correct, and unambiguous. Any typographical or grammatical errors should be corrected at revision, so please note any specific errors here.

Reviewer #1: Yes

Reviewer #2: Yes

Reviewer #3: Yes

Answer: All minor typos and grammatical errors pointed out are corrected and we have carefully reviewed the entire manuscript and corrected errors again.

(6) Review Comments to the Author

Please use the space provided to explain your answers to the questions above. You may also include additional comments for the author, including concerns about dual publication, research ethics, or publication ethics. (Please upload your review as an attachment if it exceeds 20,000 characters)

Reviewer #1: The case fatality rate (CFR), a key indicator for a communicable disease, is used to evaluate the disease severity and develop the control policy. The accurate calculation of the CFR is very important for the evaluation of disease control and treatment. The calculation methods of the infectious disease have been well studied in the past, and can be used in a new infectious disease directly. At present, the CFR of COVID-19 has been studied by many countries, organizes and researchers. There are some impacting factors of the COVID-19 CFR:

1. The CFR is very strongly impacted by the control strategy and hospital resources of the country.

2. When calculating the CFR, we need to know the number of actual cases (not merely the reported ones, which are typically only a small portion of the actual ones). A sampling COVID-19 antibody screening

was conducted in New York city, USA. The results showed the actual number was 10 times the number of confirmed cases.

3. The death number also significantly varied in the different countries because of the age of COVID-19 patients.

Above results showed the accurate estimation of the COVID-19 CFR is so complicated and impacted by many factors. So the authors of this manuscript should consider more factors in their model to get closely to the actual CFR.

Reviewer #2: This paper points the underestimation property of crude case fatality rate (CFR), defined by the number of deaths among the patients with a confirmed disease due to the under reported/under collected confirmed cases and proposes an alternative real-time estimator for CFR. This paper addresses a focal problem in estimating time-sensitive quantity, CFR during a disease pandemic. The proposed estimator was applied to COVID-19 data collected in China, South Korea and the United States. There are left room in this paper to be elaborated and more tightly written. Major and minor comments can be found in the attached review.

Reviewer #3: The paper develops a new estimation method for the case fatality rate (CFR) of a disease by taking into account potential deaths among confirmed patients alive. The method can overcome the underestimation problem of the naive CFR in an ongoing pandemic. The CFR estimation that the paper deals with is a timely research topic since the proposed method can evaluate the risk of COVID-19, an acute respiratory infectious disease that has triggered a global health crisis. The idea is novel and the paper demonstrates the utility of the proposed method with real COVID-19 data in a proper manner. The paper conducts the estimation of COVID-19 CFRs in China, South Korea, and U.S. Still, the lack of testing creates the overestimation problem since many of confirmed cases are excluded from the data. This practical limit is described in Discussion.

Data availability is not well described for reproducible research. Please add a detailed description in Data Availability Section, so readers can access the data on their own. Some language used can be improved and grammatical errors should be fixed. More details can be found in the attached.

Answer: All comments appeared here can be found in detailed comments by the referee 1, 2, and 3. Please find the answers in the point-

by-point responses to the comments below.

Point-by-point responses to Referee 1's comments

We are very grateful for your careful reading and helpful suggestions.

Major comment 1. The theoretical contribution made in Theorem 1 is one main component of the paper.

(a) Please place Theorem 1 in the main text instead of Appendix, and explain the implication of the theorem.

(b) Further, Theorem 1 (i) - (ii) are for $\hat{FR}_l(t)$ while Theorem 1 (iii) is for $\hat{FR}(t)$, thus I suggest presenting them with two separate theorems, Theorem 1 and Theorem 2.

(c) p.3 lines up 8-9. The assumptions may be required for the theorems. Please restate the theorem with the assumptions.

Answer: We carefully discussed these suggestions and agreed on (b) and (c). In particular, we appreciate the comment (c), which we missed. By following the reviewer's comment, we changed (old) Theorem 1 to (new) Theorem 1 and Theorem 2 as follows:

Theorem 1. *For each stratum l, $l = 1, \ldots, L$, and given $N_l(t)$, assume $(C_l(t), D_l(t), U_l(t)) \sim \text{Multinomial}(N_l(t), (p_c(l,t), p_d(l,t), p_u(l,t)))$, and $p_u(l,t) \to 0$ as $t \to \infty$. Then,*

(i) $E(\widehat{\text{FR}}_l(t)) = p_d(l,t) + p_d(l,t)p_u(l,t)\frac{N_l(t)-1}{N_l(t)}$, and $\widehat{\text{FR}}_l(t)$ ($l = 1, \ldots, L$) is asymptotically unbiased as $t \to \infty$.

(ii) $Var(\widehat{\text{FR}}_l(t)) = \frac{1}{N_l(t)}\eta_l(t) + \frac{1}{N_l^2(t)}\zeta_l(t)$, where (dropping (l,t) temporarily)

$$\eta_l(t) = p_d(1-p_d) + \frac{N_l(t)-1}{N_l(t)} p_d p_u \{2 - 3p_d + p_u - 4p_d p_u\},$$

and

$$\zeta_l(t) = \frac{N_l(t)-1}{N_l(t)} p_d p_u \{1 - 2p_d + 6p_d p_u - 2p_u\}.$$

Theorem 2. *Let the assumptions in Theorem 1 hold and assume that $(C_l(t), D_l(t), U_l(t))$ and $(C_\ell(t), D_\ell(t), U_\ell(t))$ are independent for $l \neq \ell$. Then, $\widehat{\text{FR}}(t)$ is asymptotically unbiased as $t \to \infty$, and*

$$Var(\widehat{\text{FR}}(t)) = \sum_{l=1}^{L} \left(\frac{N_l(t)}{N(t)}\right)^2 \left\{\frac{1}{N_l(t)}\eta_l(t) + \frac{1}{N_l^2(t)}\zeta_l(t)\right\}. \quad (1)$$

However, after careful consideration, we decided to keep these theorems in the Appendix, because the main goal of this article is to provide a new CFR estimator and corresponding module (code for calculation) for a wide usage. We think that placing Theorem 1 and Theorem 2 in the Appendix helps all readers including ones who are not familiar with mathematical terms to understand the main article easily, while the detailed statistical properties are still available for interested readers.

Major comment 2. What is the role of stratification? The potential deaths can be estimated under no-stratification. The numbers of cases, deaths, and cured in one population can be computed by summing up the numbers from all strata.

Answer: When an appropriate stratification factor (whose corresponding stratum-wise quantities – the numbers of confirmed, deceased, cured and quarantined patients – are available) is used in the proposed estimator, the role of stratification is that it helps to provide more accurate estimation of the case fatality rate with smaller standard error. We think that the case of South Korea (figure 3 in the main article, attached below) is a good example that shows an effect of stratification. In the figure, we presented daily updated 1) naive CFRs, 2) our proposed CFR estimates without stratification, and 3) our proposed CFR estimates with age-group stratification. The second one (our proposed CFR estimates without stratification) is the case the reviewer mentioned in this comment. According to the figure, the cases with / without stratification show different CFR estimates and the difference is getting greater over time, which comes from differences in age-groups.

Major comment 3. The whole data points are not in the paper, and it is not appropriate to use "All relevant data are within the manuscript and its Supporting Information files." Please use the following language that the journal provides to describe the data availability: "The data underlying the results presented in the study are available from (include the name of the third party and contact information or URL)."

Answer: Thanks for the comment. The data availability including URL is provided now in the Supporting Information section.

Major comment 4. Some descriptions can be controversial.

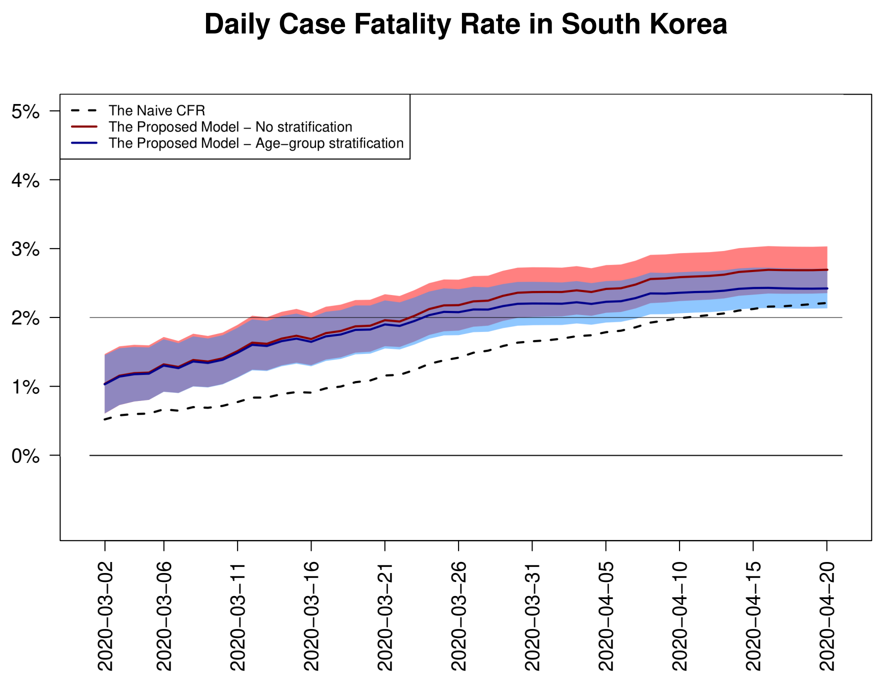

(a) p.3-4 "Because the COVID-19 disease originated in Wuhan City and ... the entire flow of the estimated case fatality rates."

(b) p.4 "Due to the late report of the missed fatalities ... the estimates of the CFR have lost trust,"

(c) p.6 "it will take a few more days or weeks for the decrease of daily CFRs"

Answer: Those sentences are revised as follows:

(a) Because the COVID-19 disease was first emerged in Wuhan City China and the first wave of the COVID-19 pandemic in China was almost over, it was meaningful.

(b) "have lost trust" → "are questionable"

(c) it may take longer than expected to see the decrease of the daily CFRs and the final convergence.

Minor comment 1. p.2 "when each stratum is heterogeneous enough" → "when we have more heterogeneous strata"

Answer: Fixed.

Minor comment 2. p.3 line 1 Patients quarantined may stay at home. So, "the patients in the hospital ($U(t)$) at time t" $\rightarrow$ "the quarantined patients ($U(t)$) at time t"

Answer: Fixed.

Minor comment 3. p.3 Are the unknown patients ($U_l(t)$) patients quarantined at time t? If so, please use the term "quarantined patients" instead of "unknown patients".

Answer: Fixed.

Minor comment 4. p.3 line 4 "the numbers of confirmed, cured, deceased and unknown" $\rightarrow$ "the numbers of confirmed, cured, deceased and unknown patients"

Answer: Fixed.

Minor comment 5. p.3 above eq (2) "the stratum-wise case fatality rate" $\rightarrow$ "the l-th stratum case fatality rate"

Answer: Fixed.

Minor comment 6. p.3 Define $E_u^d(l,t)$ in eq (2) like $E_u^d(t)$ on the 1st line.

Answer: Fixed.

Minor comment 7. p.3 Eq (3) may have $\hat{FR}(t) = \sum \frac{N_l}{N} \hat{FR}_l(t)$, which describes a relationship between the stratum-wise CFRs and the overall CFR.

Answer: Fixed.

Minor comment 8. p.3 "the proposed estimators have a consistency, that converges to" $\rightarrow$ "the proposed estimator has consistency such that it converges to."

Answer: Fixed.

Minor comment 9. p.3 "Though a duration of a disease epidemic" $\rightarrow$ "Though duration of the epidemic"

Answer: Fixed.

Minor comment 10. p.4-5 What are the CI for the naive CFRs, which are 2.17%, and 2.21% in the China and South Korea examples?

Answer: Added in the manuscript.

Minor comment 11. p.4 lines up 2-6 My understanding is that the sentence "Since the infected cases were explosively ... and patient's status changes." was added to emphasize the practical merit of the proposed method over the other existing methods requiring more pieces of data. If so, please elaborate on this. Or, the sentence may be entirely deleted as this is also explained in Introduction.

Answer: Thanks for the comment but the meaning of those sentences is a little different from the reviewer's understanding. Rather than emphasizing the merit of the proposed method over the other existing methods, the mentioned sentences are added to explain a practical reason (limitation) that we use the data from **Mar 2nd** to show an application example of the proposed method for the case of South Korea, even though the first (confirmed) case of COVID-19 was reported on Jan 20 in South Korea.

Minor comment 12. p.5 The comment that "there has been no dramatic change in daily estimated CFRs of all three estimates" seems to contradict the following comment that "the naive CFR is always outside ... of the proposed estimates".

Answer: These two sentences explain different findings as follows:

The first sentence describes an observation that NO 'dramatic change' of the estimated CFRs on a daily basis, such as the fluctuations on late January for the case of China (Fig 1) or the sharp increase and decrease on early March for the case of the United States (Fig 5), is found in Fig 3. Because such a steep change on a curve of CFRs estimated on a real-time basis happens in the early stage of the epidemic, we could find a similar shape in Fig 3 if the data before March 2 was available.

On the other hand, the second sentence explains the situation that it is still far from the final stage of the epidemic (convergence made) in South Korea because the naive CFR is significantly different from the proposed estimate on everyday in Fig 3.

To make the message clearer, we added the term 'over time' in the first sentence: "there has been no dramatic change over time in daily estimated CFRs of all three estimates".

Minor comment 13. p.5 "See that there has ... in those young age groups ... in the elderly age groups." $\rightarrow$ "There has ... in the young-age groups ($<$ 50 years) ... in the old-age groups (geq 50 years)." or something similar.

Answer: Fixed.

Minor comment 14. p.5 lines up 3-11 "By attending an assembly of the cult held in Daegu ... cult, including people without a symptom of the disease." seems unnecessary. How about deleting them?

Answer: The sentences are now deleted.

Minor comment 15. p.6 line 4 There are much more confirmed and deceased cases in USA as of today. Please update the statistics accordingly.

Answer: We fixed the time-point as April 20 for all examples (+ situation summary in the Introduction section). To make this clear, we changed the sentence as follows:

the numbers of confirmed and deceased cases have exceeded 700 thousand and 37 thousand, respectively, ***as of April 20, 2020,*** *both are now the most cases in the world.*

Minor comment 16. p.7 "Recall that we mentioned ... have been included. Nevertheless," seems redundant. How about deleting the sentences?

Answer: Fixed.

Minor comment 17. p.7 "collaboration with ..." → "integration with"

Answer: Fixed.

Minor comment 18. p.8 proof for Theorem 1. The use of X_1, X_2, X_3, and n distracts reading rather than making presentation nicer. How about keeping use of $D_l(t)$, $U_l(t)$, $C_l(t)$ and $N_l(t)$?

Answer: Fixed.

Minor comment 19. p.8 lines up 1-2 "Then (i) ...$\frac{n-1}{n}p_1p_2$." does not read well, and should be polished.

Answer: A sentence that explains the meaning is added. Please see the revised manuscript.

Minor comment 20. Please fix grammatical errors.

(a) "disease epidemic" and "disease pandemic" appear several times. Please correct them with "epidemic" and "pandemic", respectively.

Answer: Fixed.

(b) I see "that" is used instead of "which". For example, on p.3, "it is unknown during an epidemic, that makes reasonable to use". Please replace "that" by "which" where needed throughout the paper.

Answer: Fixed appropriately.

(c) p.5 line 4 "... case of China, Our estimator ... computed daily basis" $\rightarrow$ "... case of China, our estimator ... computed on a daily basis"

Answer: Fixed.

(d) p.5 lines 4-6 "As we did ..., and as the estimator ... at each time" Here, "as" appears twice. Please consider paraphrasing it.

Answer: Fixed.

(e) p.5 "this phenomenon, the group ... degree, is highly" $\rightarrow$ "this phenomenon that the group ... degree is highly"

Answer: Fixed.

(f) p.6 line up 4 "a modification of the naive CFR that allowed" $\rightarrow$ "a modification of the naive CFR that allows".

Answer: Fixed.

(g) Please replace "greater" with "higher" wherever needed.

Answer: Fixed.

Point-by-point responses to Referee 2's comments

We sincerely appreciate for your comments.

Major comment 1. It is unclear that the proposed estimator adjusts for late reported/collected deaths from quarantined patients or adjusts for unreported/uncollected deaths.

(a) Are all the quantities $N(t)$, $C(t)$, $D(t)$, $U(t)$ and $E_u^d(t)$ are cumulative number of events up to time t? If so, what factor/process makes $D(t)$ and potential death from $U(t)$ are mutually exclusive? A relevant question is whether $E_u^d(s)$ can be included in $N(t)$ for $t > s$?

(b) The clear classification for $D(t)$ should be described. For example, does $D(t)$ include only deaths primarily caused by COVID-19 and certified by physicians at hospitals? What deaths is $E_u^d(t)$ expected to cover?

Answer: The proposed estimator and the naive CFR (and other estimators of CFR also) deal with quantities from patients whose diagnoses of the disease (COVID-19) were positive (in other words, patients who were confirmed to be infected the disease). The purpose of the proposed estimator is to adjust for the bias (underestimation) of the naive CFR by considering future deaths from quarantined patients. The proposed estimator does not deal with unreported/uncollected deaths and we think that the definition of the 'case fatality rate' makes it clear.

(In Introduction section, on the second paragraph line 1, we stated *The case fatality rate (CFR) is one of the important epidemiological measures of disease severity, and is the proportion of deaths among the cases of the disease.*)

(a) Yes, all the quantities are cumulative number of events up to time t and satisfies $N(t) = C(t) + D(t) + U(t)$, i.e., the total number of confirmed cases up to time t contains the total numbers of cured, deceased, and quarantined cases which are mutually exclusive.

Recall that a quarantined patient is not 'cured' nor 'died' at the given time t. The decision will be made on sometime s, $s > t$. Since $E_u^d(t)$ is defined as the expected count of deaths among $U(t)$, it is clear that $D(t)$ and $E_u^d(t)$ are mutually exclusive.

$E_u^d(s)$ for $s < t$ is, of course, included in $N(t)$ because $N(t)$ is the cumulative number of confirmed cases up to time t. However, there is no need to consider $E_u^d(s)$ at time t. Recall by the definition that $E_u^d(s)$ is calculated from $U(s)$. All patients contained in $U(s)$ are also contained in $N(t)$ but may not in $U(t)$ because some of patients in $U(s)$ can be cured or died.

(b) The proposed estimator is mainly designed to estimate the case fatality rate using restricted information – the cumulative numbers of confirmed, cured, deceased and quarantined patients (in each stratum) up to time t. Since $N(t) = C(t) + D(t) + U(t)$, it is clear that all deceased patients are confirmed to be infected COVID-19 but we don't know all the deaths are primarily caused by the disease. Some deaths may be caused by complication developed by underlying diseases but when we deal with the quantity $D(t)$ we treat such deaths to be also caused by COVID-19. Though we exclude a possibility that some deaths can be caused by unrelated reasons such as a car accident, we believe that such cases take very small part.

Major comment 2. Are quarantined patients counted for $U(t)$ hospitalized patients? Its unclear whether $U(t)$ includes self-quarantined patients at home because $E_u^d(t)$ denotes the estimated count of deaths among the patients in the hospital $(U(t))$ at time t. If $U(t)$ excludes self-quarantined patients at home, why were such patients excluded?

Answer: The total number $N(t)$ includes all disease-confirmed patients (diagnoses for COVID-19 were positive) regardless they are in the hospital or self-quarantined. Since the proposed method estimates the case fatality rate based on the quantities – the numbers of confirmed, cured, deceased patients (up to time t) – obtained from the official reports, $U(t)$ is determined by $N(t) - C(t) - D(t)$. This means that $U(t)$ contains all quarantined patients including self-quarantined patients at home if those patients are confirmed to be infected COVID-19 (if they are included in $N(t)$).

All terms that may cause confusion are revised accordingly.

Major comment 3. Figures 2, 4, 6 provide interesting summary information but it does not additionally show any new property of the proposed estimator compared to Figures 1, 3, 5. If there exist several stratification factors in the data, it would be more interesting to see what stratification factors are more important and how finer stratifications would be proper.

Answer: Thanks for the good suggestion. However, utilizing more stratification factors is highly related with data availability. For the cases of China and the United States, we could not find available data to use stratification factors except province or state. For the case of South Korea, a few more stratification factors such as gender and region could be used but those factors could not be used simultaneously (joint table was not available). Hence, we only used the age-group for stratification that provided the most interesting result.

Minor comment 1. In lines 5 and 6 from the bottom of page 1 (Method section), Are $N(t)$; $C(t)$; $D(t)$; $U(t)$ cumulative before time t? If so, use a clear expression, for example, "by time t or up to time t" rather than "at time t" in "Suppose $N(t)$; $C(t)$; $D(t)$; $U(t)$ be the cumulative numbers of the infected, cured, deceased, and quarantined patients at time t."

Answer: Fixed.

Minor comment 2. In lines 4 and 5 from the bottom of page 1 (Method section), Grammar error: "are" to "is" in "The final turnout of the quarantined patients are unknown."

Answer: Fixed.

Minor comment 3. Lines 4 and 5 on page 2, It is unclear what the number of unknown (probably quarantined patients) is available. Does it mean that the number of quarantined patients is known?

Answer: Yes, it means the quarantined patients. All related terms are fixed accordingly.

Point-by-point responses to Referee 3's comments

We sincerely appreciate for your comments.

Comment 1. There are some impacting factors of the COVID-19 CFR.

(a) The CFR is very strongly impacted by the control strategy and hospital resources of the country.

(b) When calculating the CFR, we need to know the number of actual cases (not merely the reported ones, which are typically only a small portion of the actual ones). A sampling COVID-19 antibody screening was conducted in New York city, USA. The results showed the actual number was 10 times the number of confirmed cases.

(c) The death number also significantly varied in the different countries because of the age of COVID-19 patients.

Above results showed the accurate estimation of the COVID-19 CFR is so complicated and impacted by many factors. So the authors of this manuscript should consider more factors in their model to get closely to the actual CFR.

Answer: We think that this comment supports the advantage of the proposed estimator that adapts a stratification. We agree with the comment that considering more factors will help to estimate the actual CFR more accurately. However, using more factors is allowed only when corresponding data is available. The proposed estimator is designed to maximally use available information under restricted situation. So, when the quantities – the number of confirmed, cured, deceased and quarantined patients – are available for more factors (especially, the control strategy and hospital resources), the proposed estimator will be able to be immediately used.

Regarding the second comment above, yes, it is also important to know the number of actual cases and corresponding fatality rate to describe the risk of an epidemic such as COVID-19. However, when we consider the 'case' fatality rate, which is also an important indicator of risk of a disease, only the confirmed cases (deaths are counted from the confirmed cases) are dealt. Please find the different usage of terms "case fatality rate" and "infected fatality rate" in `https://www.nytimes.com/2020/07/04/health/coronavirus-death-rate.html`.
