# Supplementary material for: Estimation of the case fatality rate based on stratification for the COVID-19 outbreak
Source: PLoS One. 2021 Feb 22;16(2):e0246921. doi: 10.1371/journal.pone.0246921 (PMC7899354; doi:10.1371/journal.pone.0246921)
Supplement: Attachment — Submitted filename: PONE-D-20-13762_R1_review.pdf [file pone.0246921.s004.pdf]

Review on "Estimation of the case fatality rate based on stratification for the COVID-19 outbreak"

PONE-D-20-13762 R1

August 23, 2020

1. Abstract line 10 "reported quantities , the numbers" → "reported quantities, the numbers"

2. This is a follow-up comment to the response for **Major Comment 2** of the first review report. I think authors may highlight that the SE of the stratified CFR is smaller than that of the naive CFR in the case of South Korea because it may empirically show higher accuracy of the stratified CFR. If smaller error of the proposed estimator is also supported by theory, please mention it.

3. p.5 lines 18-19 I think the first percentage and CI is for the naive CFR while the second ones are for the stratified CFR.

4. This is a follow-up comment to the response for **Minor Comment 11** of the first review report. How about eliminating "Since the infected cases were ... patient's status changes. Instead, "? Or, authors may use their response to the comment: "We use the data from Mar 2nd ... as the MOHW stopped data publication from Feb 18 - Mar 1, and resumed on Mar 2 ...".

5. This is a follow-up comment to the response for **Minor Comment 12** of the first review report. Authors may elaborate on "no dramatic change". For example, "no dramatic change unlike the case of China, where we see fluctuations in late January."

6. This is a follow-up comment to the response for **Minor Comment 15** of the first review report. Please update the numbers in Introduction lines 3-4 for April 20, 2020 to have consistency.
