# Supplementary material for: Estimation of the case fatality rate based on stratification for the COVID-19 outbreak
Source: PLoS One. 2021 Feb 22;16(2):e0246921. doi: 10.1371/journal.pone.0246921 (PMC7899354; doi:10.1371/journal.pone.0246921)
Supplement: Attachment — Submitted filename: Response_to_Reviewers.pdf [file pone.0246921.s005.pdf]

Revision Report (PONE-D-20-13762) - Round 2

Byungwon Kim, Seonghong Kim, Woncheol Jang, Sungkyu Jung, and Johan Lim

September 25, 2020

We appreciate the Academic Editor and the referees for their thoughtful comments on our revised manuscript, titled "Estimation of the case fatality rate based on stratification for the COVID-19 outbreak". In this second revision, one reviewer gave us a few minor comments as a follow-up to the previous comments, and we have carefully followed the suggestions and made appropriate changes. Please find our point-by-point responses to the reviewer's comments.

Point-by-point responses to the Reviewer #3's comments

We are very grateful for your careful reading and helpful suggestions.

1. Abstract line 10 "reported quantities , the numbers" → "reported quantities, the numbers"

   Answer: Fixed.

2. This is a follow-up comment to the response for Major Comment 2 of the first review report. I think authors may highlight that the SE of the stratified CFR is smaller than that of the naive CFR in the case of South Korea because it may empirically show higher accuracy of the stratified CFR. If smaller error of the proposed estimator is also supported by theory, please mention it.

   Answer: Thanks for the comment. Unfortunately, there is no theoretical and empirical clue that the standard error (SE) of the stratified CFR estimator is always smaller than that of the naive CFR. When comparing the stratified CFR estimator with the unstratified CFR estimator (both are our estimators, not the naive CFR), we have empirically found that the SE of the stratified CFR estimator is slightly smaller than that of the unstratified CFR estimator. However, it is

not in general true for the comparison with the naive CFR. There is a counter example in our manuscript; please see p.5, the paragraph right above the caption of Fig 3. On April 20, the 95% confidence interval of our stratified CFR is (2.13%, 2.71%) whose length is 0.58 while the 95% confidence interval of the naive CFR is (1.93%, 2.49%) whose length is 0.56.

3. p.5 lines 18-19 I think the first percentage and CI is for the naive CFR while the second ones are for the stratified CFR.

   Answer: Those percentages are correctly given; the first one is for the stratified CFR estimator and the second one is for the naive CFR. By the definition of our estimator, it must be greater than or equal to the naive CFR.

4. This is a follow-up comment to the response for Minor Comment 11 of the first review report. How about eliminating "Since the infected cases were ... patient's status changes. Instead, "? Or, authors may use their response to the comment: "We use the data from Mar 2nd ... as the MOHW stopped data publication from Feb 18 - Mar 1, and resumed on Mar 2 ...".

   Answer: We changed the sentences on page 4 as follows:

   We use the data from Mar 2 as the Korea center for disease control and prevention (KCDC) and the ministry of health and welfare (MOHW) stopped data publication from Feb 18 to Mar 1, and resumed on March 2.

5. This is a follow-up comment to the response for Minor Comment 12 of the first review report. Authors may elaborate on "no dramatic change". For example, "no dramatic change unlike the case of China, where we see fluctuations in late January."

   Answer: Thanks for the suggestion. We have corrected the sentence on page 5 as advised by the reviewer.

6. This is a follow-up comment to the response for Minor Comment 15 of the first review report. Please update the numbers in Introduction lines 3-4 for April 20, 2020 to have consistency.

   Answer: The numbers are updated. See page 1.
